# Intersubjectivity in schizophrenia: life story analysis of three cases

**DOI:** 10.3389/fpsyg.2014.00100

**Published:** 2014-02-12

**Authors:** Leonor Irarrázaval, Dariela Sharim

**Affiliations:** ^1^Centro de Estudios de Fenomenología y Psiquiatría, Facultad de Medicina, Universidad Diego PortalesSantiago, Chile; ^2^Escuela de Psicología, Facultad de Ciencias Sociales, Pontificia Universidad Católica de ChileSantiago, Chile

**Keywords:** schizophrenia, phenomenology, hermeneutic, intersubjectivity, life stories, clinical psychology

## Abstract

The processes involved in schizophrenia are approached from a viewpoint of understanding, revealing those social elements susceptible to integration for psychotherapeutic purposes, as a complement to the predominant medical-psychiatric focus. Firstly, the paper describes the patients’ disturbances of self-experience and body alienations manifested in acute phases of schizophrenia. Secondly, the paper examines the patients’ personal biographical milestones and consequently the acute episode is contextualized within the intersubjective scenario in which it manifested itself in each case. Thirdly, the patients’ life stories are analyzed from a clinical psychological perspective, meaningfully connecting symptoms and life-world. Finally, it will be argued that the intersubjective dimension of the patients’ life stories shed light not only on the interpersonal processes involved in schizophrenia but also upon the psychotherapeutic treatment best suited to each individual case.

## INTRODUCTION

Pathological experiences are usually described as phenomena that are divorced from the life context in which they are manifested. Nevertheless, in the field of phenomenological psychopathology, symptoms have traditionally been considered from a more comprehensive perspective: they are embedded in the person’s life thus their contents and meanings can only be understood within the context of that life. In themselves “unhistorical,” symptoms become connected meaningfully only within the comprehensive picture of the patient’s life as a whole (Jaspers, 1997).

An even stronger argument could be made to the effect that “no mental illness can be diagnosed, described, or explained without taking account of the patients’ subjectivity and their interpersonal relationships” (Fuchs, 2012, p. 342). It is clear that psychopathological manifestations cannot simply be reduced to the workings of the nervous system (Fuchs, 2011). For that reason, the recommendation here would be not to establish linear or “cause/effect” relationships, but to approach mental illnesses with the notion of a “circular” mode of causality, regarding their emergence from subjective, neural, social, and environmental influences continuously interacting with each other (Fuchs, 2012).

Contemporary psychopathological phenomenology regards schizophrenia as a paradigmatic disturbance of embodiment and intersubjectivity (Dörr, 1970, 1997, 2005, 2011; Blankenburg, 2001, 2012; Fuchs, 2001, 2005, 2010a; Sass and Parnas, 2003; Stanghellini, 2004, 2009, 2011). From this approach, it seems appropriate to use methods that attempt to characterize not only the patients’ symptomatic disturbances but also the interpersonal processes involved, broadening the scope of exploration to areas not taken into account in the criteriological manuals of diagnostic systems Diagnostic Statistical Manual of Mental Disorders (DSM) and International Classification of Deseases (ICD) (Fuchs, 2010b).

This paper presents the life story analysis of three cases that form part of the corresponding author’s doctoral dissertation entitled “Study of disorders of the pre-reflexive self and of the narratives of first admitted patients with schizophrenia” (unpublished), covering a total of 15 patients with schizophrenia during their first psychiatric hospitalization.

The processes involved in schizophrenia are approached from a viewpoint of understanding, revealing those social elements susceptible to integration for psychotherapeutic purposes, as a complement to the predominant medical-psychiatric focus. Firstly, the paper describes the patients’ disturbances of self-experience and body alienations manifested in acute phases of schizophrenia. Secondly, the paper examines the patients’ personal biographical milestones and consequently the acute episode is contextualized within the intersubjective scenario in which it manifested itself in each case. Thirdly, the patients’ life stories are analyzed from a clinical psychological perspective, meaningfully connecting symptoms and life-world. Finally, it will be argued that the intersubjective dimension of the patients’ life stories shed light not only on the interpersonal processes involved in schizophrenia but also upon the psychotherapeutic treatment best suited to each individual case.

Here, “life-world” refers to the person’s subjectively experienced world, which emerges in the process of conceiving one’s self and the others through a history of social interactions (Husserl, 1970; Schutz and Luckmann, 1973; Varela, 1990; Varela et al., 1991; Maturana and Varela, 1996).

## MATERIALS AND METHODS

### STUDY DESIGN

The study was developed within the qualitative paradigm, it being an explorative–descriptive type of study. This type of studies proceeds with inductive logic: in other words, both hypotheses and analysis categories are developed as the study progresses, and emerge from the data itself (Danhke, 1989 quoted in Hernández et al., 2003).

The so-called “critical case sampling” criteria was used, where the interest in an in-depth approach to the phenomena means working with few cases, with representativeness not being of key importance for these purposes. Thus, the significance and understanding emerged by qualitative inquiry have more to do with the richness of the cases chosen and also with the observational and analytical abilities of the researcher, rather than with size of the sample (Patton, 1990; Schwartz and Jacobs, 1996; Creswell, 1998).

### PARTICIPANTS

The broad research covered a total of 15 patients with schizophrenia during their first psychiatric hospitalization. All of them were males, aged between 18 and 25. Additional inclusion criteria were the following: (1) accessibility to the sample, (2) homogenous sample (Halbreich and Kahn, 2003), and (3) earlier first onset and higher risk of developing schizophrenia in men (Aleman et al., 2003).

The three cases were selected due to the variety of subtypes to illustrate the interpersonal processes involved in schizophrenia, taking the intersubjective dimension of the patients’ life stories into consideration. Cases 1, 2, and 3, as they appear in the paper, correspond to patients with diagnoses of disorganized-type, paranoid-type, and catatonic-type schizophrenia, respectively.

### INSTRUMENTS

#### In-depth interviews

In-depth interviews were used to gather qualitative data from the first encounter with the patients and from their life stories. These interviews had open questions aimed at allowing for a natural manifestation of the patients’ accounts. For the first encounter, the recommendations on interviews for the phenomenological diagnosis of schizophrenia were taken into account (Dörr, 2002), and clinical biographical focus criteria were used to perform the life story interviews (Sharim, 2005).

#### Positive and Negative Syndrome Scale

The Positive and Negative Syndrome Scale (PANSS; Kay et al., 1987) is a rating scale used for measuring symptom severity of patients with schizophrenia. The name refers to the two types of symptoms: positive, which refers to an excess or distortion of normal functions (e.g., hallucinations and delusions), and negative, which represents a diminution or loss of normal functions.

#### The Examination of Anomalous Self-Experience

The Examination of Anomalous Self-Experience (EASE; Parnas et al., 2005) is a semi-structured interview for the phenomenological examination of disorders of the pre-reflexive self, postulated as early markers or basic phenotype of the schizophrenic spectrum (Raballo et al., 2011). The EASE explores a variety of anomalous self-experiences, which typically precede the onset of positive symptoms and which also often underlie negative and disorganized symptoms (Parnas and Handest, 2003).

### PROCEDURES

Data gathering was performed by means of semi-structured interviews, which are characterized by the use of eminently “open” research questions. Less structured methods allow for the emergence of ideographic descriptions, personal beliefs and meanings, focusing on “how” the psychological processes occur (Barbour, 2000).

Five encounters with the patients were carried out. These encounters were coordinated throughout the three following phases:

**Phase I:** A first encounter to record the patients’ accounts of the disturbances of self-experience and body alienations manifested in the acute episode (30–45 min interview carried out 1–2 weeks after hospitalization), following the confirmation of the diagnosis of schizophrenia in accordance with expert judgment and the standard diagnostic criteria of DSM-IV-R (American Psychiatric Association, 2003) and ICD-10 (OrganizaciónMundial de la Salud, 2003).

**Phase II:** Two subsequent encounters to carry out the EASE (Parnas et al., 2005; 30–45 min per interview carried out 1 month after hospitalization), when patients did not score with “positive” symptomatology on the PANSS (Kay et al., 1987).

Note: The results of Phase II of the broad research have not been included in this paper. The results from the EASE exploration will be published in a complementary paper focused on basic self-disorders entitled “The lived body in schizophrenia” (in preparation).

**Phase III:** Finally, two further encounters were held to perform the life story interviews (30–45 min per interview carried out 1–2 months after hospitalization). The first encounter started with the open instruction “tell me about your self,” “tell me about your life,” while the second one was focused mainly on the patients’ significant social interactions and personal meanings, also including their first image in life, their early dreams (hopes), their self-definition, and their expectations about the future.

All the interviews were recorded on video and fully transcribed for subsequent analysis. Extracts of the patients’ accounts were kept literally in quotes.

### ANALYSIS

#### First encounter (Phase I)

The patients’ accounts of the disturbances of self-experience and body alienations manifested in the acute episodes were summarized in corresponding descriptions containing the essential structure of the transcripts, which were obtained with the “Descriptive Phenomenological Method in Psychology” (Giorgi, 2009), by following five steps: (1) the researcher reads the entire transcript in order to gain an overall sense, (2) the same transcript is then read more slowly, and underlined every time a transition in meaning is perceived, providing a series of units constituting meaning, (3) the researcher then eliminates redundancies and clarifies the meaning of the units, connecting them together to obtain a sense of the whole, (4) the arising units are expressed essentially in the language of the subject, revealing the essence of the situation for him, and finally, (5) there is the summarizing and integrating of the achieved understanding in a description with the essential structure of the transcript.

#### Life story interviews (Phase III)

The criteria of the clinical biographical focus were considered in the life story analysis, which are part of the so-called “clinical human sciences” paradigm (Legrand, 1993; Sharim, 2005, 2011). This approach stresses the life story method, in which the clinical dimension is constantly present, working primordially on singularity: case-by-case, story-by-story.

At the same time, the examination of singularity and heterogeneity of individual situations allows the progressive appearance of common processes that structure behavior and organize these situations (Sharim, 2005, 2011; Cornejo et al., 2008). This method highlights the role of the subject in recounting his life story, giving the possibility to analyze the reciprocal relationship between the subject’s determination by his history and his potential to create his own existence (De Gaulejac, 1999; De Gaulejac et al., 2005).

The in-depth analysis of the life stories was developed under a course guided by the co-author of this paper. The course was called “Hermeneutic analysis of biographical material for the study of patients with schizophrenia” and took place during one academic semester at the Catholic University of Chile. The analysis focused on the personal meanings (Fuchs and De Jaegher, 2009) by following the patients’ history of significant social interactions.

Therefore, the transcripts were analyzed by peer researchers (corresponding author and co-author of this paper) both clinical psychologists with a specialty in psychotherapy. To avoid bias each researcher previously made a separate analysis and then met for the co-analysis, ensuring with this procedure the validity of the qualitative research (Maxwell, 1996; Morrow, 2005; Fischer, 2009).

Firstly, an individual (case-by-case) in-depth analysis of each narration using a hermeneutic approach was carried out. In this analysis each life story was re-constructed, carrying out a thematic and chronological ordering, which enabled the identification of “biographical milestones,” as well as the analytical axes in each life story. Second, a cross-sectional analysis was carried out contemplating the stories all together, revealing the differences, similarities, and shared structural dimensions.

### ETHICAL ISSUES

The broad research, covering 15 patients with schizophrenia during their first psychiatric hospitalization, was regarded as entailing no physical, psychological, or social risks for the subjects involved, based on the Declaration of Helsinki principles, the Council for International Organizations of Medical Sciences (CIOMS) 1992 International Ethical Guidelines for Biomedical Research Involving Human Subjects, and the 1996 International Conference on Harmonisation (ICH) Good Clinical Practice guidelines, by the following Ethics Committees: (1) Research into Human Beings Ethics Committee of the University of Chile’s Medical Faculty, dated January 19, 2011. (2) Ethics Committee Research of the Psychiatric Hospital, dated August 2, 2012. (3) Ethics Committee Research of the North Metropolitan Health Service (Santiago, Chile), dated August 16, 2012.

The Ethics Committees also approved the patients’ and their tutors’ (legal representatives) consent documents. In this regard, the following ethical aspects were taken into account: (1) consent was informed and obtained from the patients’ tutors by the attending doctor at Phase I of the study, considering that as a patient affected by an acute episode of schizophrenia, his competence or capacity is diminished and he must be authorized to participate. (2) Consent was obtained directly from the patients at Phase II of the study. (3) Pseudonyms were employed to protect the identity of the patients and ensure confidentiality (internal codes were used for each patient to replace their original names).

Note: Careful attention was paid in this paper to the protection of the patients’ anonymity. Identifying information such as dates, locations, hospital numbers, etc., was avoided.

## RESULTS

### INDIVIDUAL ANALYSIS (CASE BY CASE)

#### Case 1

Santiago (Santi) is an 18-year-old patient, diagnosed with disorganized-type schizophrenia. He has completed 8 years of basic school education. His father died of cancer 1 month before his hospitalization: until then, he lived with him and his two brothers. He is the middle brother. The patient’s mother left home when he was 12 years old.

***First encounter***. A first interview was carried out after 2 weeks of hospitalization. In this encounter, the patient indicates that although he considers himself to be a “normal” person, begins to recognize a “*repetitive failure*.” It is primarily the mediating process of thinking that has become the main impediment in this case.

The patient indicates that he hears voices, which are as if his own thoughts were repeated inside his head, like an echo, “*as if I was reading them aloud but with my mouth closed*.” Most of the voices repeat meaningless things that he does not understand. He also hears voices on the radio, repeating what he is thinking: these are voices of unknown people who seem to be talking to him. Additionally, it sometimes seems to him that some television personalities repeatedly say things to him, all sorts of non-sense. He does not know how or why they do.

There are periods in which the “repetitive failure” intensifies, to the extent that it prevents him leaving home, and that only by going to bed to sleep is he able to take a break from these thoughts. This has made it difficult for him to progress with his studies or concentrate. He feels that this situation is annoying for him and is harmful because he cannot live a normal life.

At first, the patient figured it was sort of a game, playing with the voices and thoughts, but he could not control it, he could not stop it, he kept on playing. This was sometimes unbearable for him, and has even made him want to hang himself.

***Biographical milestones***. The life story interviews were carried out after 2 months of hospitalization. The patient was receiving the usual pharmacological treatment and had recently completed 12 electroconvulsive therapy sessions.

“My mum left me when I was 12”

Santi begins his account by indicating that he has had a hard life. He refers to his parents’ divorce, and particularly to when his mother left him alone with his brothers when he was 12. His mother moved away from the city and got married again. “*It was very hard, when she wasn’t there and we lacked a mother’s love*.”

In addition to being angry with his mother when she left home, Santi also points out that he did not get on with her as a child. He remembers that she used to get very annoyed with him when he and his father sometimes made fun of her.

The mother returned after 2 years for her children. Santi’s brothers agreed to go with her, but he preferred to remain with his father. At the age of 14, he was living alone with his father. However, the brothers returned 2 years later, when he was 16, due to the serious situation with the mother’s new husband, who beat them.

Santi states that he got on well with his brothers; they had an affectionate relationship, one of friends, between them. They helped each other out and shared the housework between them.

“I died in high school”

Santi acknowledges that a significant change took place in his life at school. As a young child, he was a very good pupil and wanted to study medicine, but at the age of 12 he lost interest in his studies, skipped school, and began taking drugs. He had to repeat the last school year twice due to absenteeism. He liked the typical tools of the medical trade and wanted to have a stethoscope. “*Now that I have them here* (at the psychiatric hospital), *I ask myself, why can’t I, if everyone else can?*”

He stopped taking drugs at the beginning of this year and returned to his studies. He wanted to study accountancy to earn money. He had recently started the first year of high school when he was hospitalized.

“My dad passed away recently”

Santi states that his first memory is one of being with his family, when he was 7. It is a memory of the time when they were still living with their mother. He recalls it was his father who took them to a pretty square at the center of the city. “*Nice memories, everything was nice with my dad.*”

The father worked in the public sector and had taken early retirement, the reason for which is unknown. He did not remarry or have a relationship with another woman. Santi has a very positive image of him. He describes him as hard worker, a good father and who liked to go out and play ball with him and his brothers.

Santi displays an empathetic attitude toward his father, even a certain loyalty, which is made clear when he recounts the time when his mother left home, and later when his brothers left. In fact, he decided to stay alone with his father, despite the pain caused by the separation from his mother and brothers. “*My dad went through an extremely painful time, to put it one way, he didn’t show it but, inside, he was feeling bad*.”

The father passed away 3 months ago, from cancer, at the age of 65. He became ill a month before dying, and had immediately told his sons of his disease, so they were aware of how much longer the doctor had given him. The father was hospitalized at the time of his death.

Santi recognizes that he was very attached to his father, he states that “*even too much*.” He realizes that he still has not gotten over the death of his father, “*because of my illness, I still have not gotten over it. I haven’t realized what it all really means*.”

“*I see the future as nothing*”

Since the last 4 years, Santi has been becoming more and more distanced from the world, to the point where he is extremely isolated. He has no friends, does not study or work, takes no part in social activities and has not embarked on any romantic relationship.

During the week, he helped with some household chores, such as making lunch. Nor did he do anything special during the weekend, except go out to the square with his brother. He spent a lot of time in his room playing on his PlayStation. “*I see the future as nothing, the way I’m going. Not doing anything, not studying, because where will I get like this? It’s looking bad, isn’t it? I’m worried*.”

***Life story analysis***. The patient took part in the interviews without any problems. He appeared interested in obtaining more information on his state of health and motivated to seek help to secure a speedy discharge. He interrupted the interviews on a number of occasions to ask what his illness was, if it was very serious and when his attending doctor would discharge him. Generally, he appeared constantly concerned about his state and anxious to put an end to his confinement.

His life story contains a series of events that could be regarded as stressful. It is certainly possible to establish a connection between the death of his father (i.e., the patient’s state of grief) and the emergence of the first acute episode, and also to identify his mother’s leaving home as the crucial biographical milestone in the development of the prodromal stage of schizophrenia. Somehow, the sense of abandonment in the world has come to dominate the patient’s life.

The scale of the emotional impact of the recent loss of a father is obvious: nevertheless, the patient at no time displays any signs of sadness and does not cry. Instead of a spontaneous emotional expression, he rationally discerns the seriousness of the situation and like a “witness” he testifies the tremendous impact this must have on his life.

He manifested an initial perplexity, conveyed with a degree of humor, in light of the apparent oddness and incomprehensibility of the account of his anomalous experiences (“the repetitive failure”). Nevertheless, although he recounts sad events in his life, any actual sadness can only be assumed. To put it one way, it is possible to “intuit” the patient’s suffering, through the loneliness, abandonment and lack of support in his life, rather than by means of an explicitly emotional manifestation on his part.

The patient notices the paradoxical situation involved (of being hospitalized) when he states that he regards himself as a “normal” person, except for his “repetitive failure.” Far from merely being a game, as he previously regarded it, it is now given the name of schizophrenia, a diagnosis that defines him as a seriously ill patient and justifies his compulsory commitment to a hospital. This has led him to realize that what is happening to him is not socially acceptable, and is thus regarded as more serious in his own judgment.

#### Case 2

Angel is a 22-year-old patient, diagnosed with paranoid-type schizophrenia. He has 11 years of basic school education and lives with his parents and the eldest of his three sisters. He is the youngest of the siblings and the only brother. His family are evangelical Christians.

***First encounter***. A first encounter was carried out a week into his hospitalization. The patient has not been able to find a convincing explanation for the fear he feels, which he recognizes as his major impediment. He thinks he could be delivered over to the Tribulation – the Tribulation is a biblical time of pain.

About 3 months ago he began to feel persecuted by people. His house was the only place he felt safe, but for a few weeks now he has even begun to feel unsafe at home. The idea that somebody can hurt him comes from the fear he feels and he thinks that the worst thing would be that somebody kills him somehow, like stabbing him, for example. This fear is a distressing feeling, of wishing to escape, when he suddenly feels that something bad is going to happen to him.

He is quite concerned about his problem, and thinks a lot about it, and how to solve it. He wants to find a way to overcome the fear. He would like to find a “*clear and precise*” answer to what he should do, how he should live and how to face up to his fear. He wishes that the bible could tell him what to do in the Tribulation, “*if I was in that time, that it told me in light of this fear to do this or that, to face up to it, don’t be afraid, I’ll be with you*.”

***Biographical milestones***. The life story interviews were carried out 1 month into the patient’s hospitalization. He was receiving usual pharmacological treatment and his suitability for electroconvulsive therapy was being assessed.

“*When I was a kid I went to school*”

Angel woke up one night and found himself alone at home: it was very dark and he started crying. This is the earliest image that he recalls from his childhood. He also remembers that he would sometimes run up the stairs because he thought that someone, “*perhaps the bogeyman*,” was after him.

He remarks that his grades were not great but things went well for him at school. During his childhood, he felt good because he went out to play and climb trees. He also liked to fix televisions and take apart toy cars. He stresses the fact that he was more outgoing and playful as a child.

His family was always good to him, and he notes that he had a happy childhood. He was closest to his mother, as she stayed at home and was very attentive and loving toward him. His mother was of good character, and only punished him on a couple of occasions, “*because once I hit my sister with a hammer, when I was playing, and my mum punished me, she gave me a slap on the behind*.”

“My sisters were very critical of me”

Angel has three older sisters. He has had a difficult relationship with them, and particularly with the eldest. He points out that his sisters criticized him a great deal and made fun of him. Therefore, even as a child, he took great care to say the right thing, so as not to make a fool of himself and feel embarrassed.

He was not only concerned to ensure that he said the right thing, but also with his personal appearance. He was very sensitive about the comments his sisters made about him. He states that he was very shy as a child, and when he was embarrassed by something he would run away and did not want to come back.

“Then I went to high school”

At high school, Angel was unable to make friends. He notes that he changed, became less playful, less “chatty” and more reclusive. He did not play ball so much or join in with classmates as often.

He also comments that he found it difficult to appear in front of his classmates, and skipped school when he had to give a talk to the class on a subject. This got worse when he started to suffer from acne, which made him feel that people were looking at him too much and a little persecuted.

It was because of the acne that Angel began to skip school, until he stopped going completely and became totally isolated. “*By this point, the acne wasn’t as bad, but it was the fact I missed school, I skipped class a lot, I was embarrassed that I skipped school so much, and that’s why I stopped studying*.”

“*Then I went out to work. That’s when it all went wrong*”

Angel does not think that his acne is any better, but somehow he learned to come to terms with this concern. He has spent a lot of time at home, in his room playing on his PlayStation. This is what he has mostly done over the last 4 years, as he admits. “*I didn’t see anyone except for my family, not friends, because it’s a bit solitary on the PlayStation, you get closed in on yourself when you’re on it*.”**

After 4 years, Angel went out to work. He notes that it is when everything went wrong. He had spent a lot of time at home, without going out. He notes that he was perhaps unprepared to go out and experience life like that all of a sudden. It was then that he began to feel that people were after him.

“*Now, as a person*”

In adolescence, Angel wanted to be an air force pilot but he could not apply because he did not finish his studies and was under the required height – “*it came as quite a blow, but I was still interested in mechanics*.”

Angel does not have a clear vision of what the future holds, principally because he has not overcome the fear of being harmed and the thought that “somebody” will kill him, which is his most serious affliction. Nevertheless, he indicates that, if he can overcome his fear, he would like to work and study mechanics and electronics, which have been interests of his since childhood.

***Life story analysis***. The patient was very willing to take part in the interviews, although he generally appeared tired and dispirited. He seemed not to have much to say, or not to be ready to recount his story. He is of a religious disposition and a frequent reader of the Bible where, above all, he hoped to find an explanation for the problem affecting him: his fear.

His account is mainly based around the fear of being harmed, which is the subject of his delusion. He even appears, in a way, excited when talking about the problem of his fear and about the different explanations he uses to understand what is happening to him. Aside from this core problem afflicting him, his account barely touched on other aspects of his life, and he appeared to become dispirited, tired, and uninterested when moving away from the subject of his delusion.

He seems concerned that he is unable to find certainty in things, above all with regard to explaining his fear. He feels prey to a fear that is completely restrictive, and is unable to find a satisfactory explanation that would allow him to understand what is happening to him or to give a completely convincing response to overcome the situation. He is aware of the extent of the fear and the significant limitations it causes in his life, and of the lack of any clear orientation as to how to overcome it.

The patient conveys a feeling of “ontological” uncertainty or insecurity. From an early age in his life, the world (and others) acquired a sense of unreliability or threat. Shame and fear of ridicule are the predominant emotional aspects of his experience in childhood. Somehow, later on in adolescence these emotions led to the fear of persecution. Persecution progressively became a fear of being hurt until it reached the extreme point of a fear that he would be killed, which manifested itself in the first acute episode.

#### Case 3

Salvador (Salva) is a 25-year-old patient, diagnosed with catatonic-type schizophrenia. He has completed 12 years of compulsory school education and lives with his father and older brother. His parents divorced 2 years ago.

***First encounter***. The first interview was carried out when the patient had been hospitalized for close to 2 weeks. He explains that 2 years ago started with an episode of mental illness: “*I was getting cramps in the back of my brain*.” It was because of the confusion these cramps caused in his brain that he went to the psychiatrist. Then, he was diagnosed with depression and treated with medication for a year but the problem persisted.

He feels mental pressures, and indicates it is as if they squeeze his brain. His thoughts are jumbled up, all messed up with ideas. Reality gets distorted for him as well, as if he were in a constant dream. In addition, he has felt someone possessing his body and explains it as “demonic possession.” He thinks that spirits get in when someone is depressed. It is something he cannot control, something unpredictable, imminent.

The patient is worried about the state of his mental health. It worries him to “live like this,” and he feels a deep-seated desperation. He does not want to do anything and feels depressed, downcast, dispirited, and powerless. Before he was hospitalized, he wanted to committed suicide by jumping off a hill due to the desperation.

***Biographical milestones***. When the life story interviews were carried out, the patient had been hospitalized for a month and a half. He was receiving the usual pharmacological treatment.

“*My interest in religion began at the age of 8*”

Salva completed his primary education at a Christian school. He liked the religious part of school because religion was taught in a fun way. When he was a child, he used to go to church with his family. “*I liked the teachings about love, love for one another, love for one’s neighbor*.”

He points out that he was a very good student and got very good grades. He wanted to be a vet when he was a child, because he liked animals. He describes himself as a gentle, playful, brotherly, sweet boy.

“*They moved me to a worldly high school*”

The change of school had a negative impact on Salva. His performance suffered, and he went from being an outstanding student to being just an average one. He notes that students at the new school were treated more coldly.

He had wanted to be a vet since childhood but he could not go to university, as he did not pass the entrance exams. He therefore chose to study architectural drawing at a college, but did not manage to complete his first year there.

“*My mum was sweet to me when she was Evangelical*”

Salva had a good relationship with his mother as a child. He points out that his mother was very loving toward him whilst she was Evangelical. Later, however, for reasons unknown to him, she distanced herself from church. Their relationship deteriorated when he was a teenager.

He got on badly with his mother because, he explains, of their very different characters. His mother ill-treated him and frequently insulted him. This made him feel powerless. “*She was really aggressive, and punished and hit me for anything. She used to insult me in all kinds of ways, she called me mentally ill*.”

His mother also fought with his father and brother. She drank, and when she did so she became more violent.

“*I went through a lot in 2010*”

Salva states that he had his first episode of “mental illness” 2 years ago, and has not been able to work or study since then. “*I did nothing at home, just playing games on the computer; I’d play on it, football games and PlayStation. I spent a load of time doing that*.”

It was in this same year that his mother left home and his father fell ill with diabetes. His brother had had a heart attack at the end of the previous year.

His mother left home to live with a new partner, saying she wanted her independence. At first he missed her, but was also angry. He did not want to see her or be with her after she left.

Salva continued to live with his father and brother. He feels very attached to them, and is concerned about their health. He feels he has a really great father, because he has had to play a double role. He gets on well with his brother too, who he regards as a second father.

“*It’s great at church, they treat me really well*”

Salva’s current friends are evangelicals and he joins them at church. He likes going to the church because there he got to know beautiful people and had a much closer relationship with God. “*I like being in communion with God, praying, singing, that’s how I look for protection*.”

He has had four episodes of “demonic possessions,” all of which happened at church. It was at church where he was told that his bodily experiences were “possessions” and that they are somehow “normal.” However, the treatment he was given there was unsuccessful. They carried out “deliverances,” which are a way of getting the devil out the body with prayer.

At the moment, Salva does not know why these episodes have happened to him, or whether they are due to an illness, and has not even talked much about the matter with his attending doctor.

“*In the future, I want to study massage therapy*”

Over the course of the last 7 years, Salva worked on and off in a number of fields. He took jobs as a shelf stacker in a supermarket, a cleaner at a cinema and a shop assistant. His last job was 2 years ago selling fragrances in a street market.

He has remained socially isolated over the last 2 years, only keeping in touch with his evangelical friends at church sporadically. “*I’ve found it difficult to relate to people in recent years. I haven’t worked much or had much of a social life. I’ve been isolated*.”

In the future he would like to have children, a wife and work giving massages, although he realizes that he remains scared about his mental state, that he feels vulnerable.

***Life story analysis***. The patient took part in the interviews willingly, although he did appear very tired and sleepy (he was constantly yawning). The disordered thoughts persist, as do his low spirits, mental pressures and the uncertainty in the face of possible new “possessions.” He talks about himself and his life quite candidly and seems naïve, as if recounted by a small child. He speaks calmly, slowly, with little verve. It is a story with few elements told at a basic level of articulation.

He is very religious, a habitual reader of the Bible and a regular churchgoer. Now, although the episodes were “demonic possessions,” fear does not appear to be the predominant or explicit emotion: it is rather the loss of control of his bodily experiences and the unpredictable nature of these episodes that make the patient desperate. In other words, his desperation is due to his inability to once again feel normal or healthy.

He left school 7 years ago and has not developed a specific plan to carry out his life. Although he wishes to have a “normal” life, his life project faces a vacuum. However, the lack of a plan does not seem to concern him at all. Instead, what most worries the patient at present is the state of his mental health, that is, the anomalous bodily experiences he is not able to control.

It is possible to make a connection between the emergence of the first acute episode and a series of stressful events that occurred in the patient’s life at that time: his mother left home, his father fell ill with diabetes and his brother had heart problems, all in the same year. Although, the negative impact of the change in high school and the deterioration of the relationship with his mother in his adolescence are the crucial biographical milestones identified in the development prodromal stage of schizophrenia.

Besides, what the patient explains as “spirits getting into” does not seem to correspond to a typically clinical depression (as it was diagnosed initially), but rather to a severe “passivity” of his own existence, which finds concrete form in his disembodied experiences.

### CROSS-SECTIONAL ANALYSIS

The cross-sectional analysis shows that a severe disorder of intersubjectivity starts developing in early adolescence. Beginning at an early stage, the patients progressively distance themselves from the social world. This distancing becomes a structural element, a key part in the prodromal stage of schizophrenia.

It is not an active deliberate distancing, but rather an overall difficulty that hampers the living of a normal life. It implies a progressive “passiveness” of the patients’ own existence, which manifests itself not only in the disturbances of self-experience and body alienations of the acute phases, but also in the patients’ radical withdrawal from the social world.

For several years, the patients have not worked or studied, have had no social life, and have stayed shut in at home watching television or playing on their PlayStation for hours at a time. Here, it is important to notice that the acute episode occurred at a time when they were planning to return to their studies or the world of work after a number of years of extreme isolation.

It is possible to make a connection between the prodromal stages of schizophrenia and several stressful events that occurred in the patients’ lives. It is also possible to follow a continuity in the experience of vulnerability regarding the main personal meaning configured early in life: the feeling of abandonment, the fear of ridicule and the feeling of powerlessness, corresponding to Cases 1, 2, and 3, respectively.

Nevertheless, the patients’ withdrawal from the social world is what eventually leads to the manifestation of their psychosis. Somehow, in their attempts to returning to intersubjectivity, all of a sudden the patients confront themselves with their own “vulnerability” of being in the world.

Although they have some ideas about what to do in the future, the patients are insufficiently prepared, and lack a specific plan to implement them properly. Their life project faces a vacuum. This is what makes their condition so severe: there is an interruption in the patients’ normal unfolding of life.

The patients do have a concept of what a “normal life” should be (basically, to study, to have a job, to marry, and to have a family), but they do not seem to possess the factual grounding needed to deal with the world, as if they were lacking the implicit “know how” to carry out the normal life they wish to live.

It should be noted that the patients’ life stories feature a series of healthy elements or personal qualities that reflect a certain nobility of character: sensitivity, authenticity, naivety, empathy, and innocence. There does not appear to be any secondary gain associated with the symptoms.

## DISCUSSION

### KEY FINDINGS

In acute phases of schizophrenia, patients’ accounts concentrate on (or are limited to) the disturbances of self-experience or body alienations. In other words, patients’ accounts lie outside the time-space dimension of the social context and exclude personal history. Body alienation appears to be the way in which the de-subjectivized accounts find concrete form (or are materialized).

The assessment of the life stories complements the symptomatic descriptions embedding them in the patients’ life-worlds, thus incorporating a social horizon. In this way, the dimension of intersubjectivity is illustrated in the patients’ history of significant social interactions, discovering the interpersonal elements to integrate in psychotherapeutic and prevention models.

The articulation of the patients’ life stories allow to follow the patients’ progressive withdrawals from the social world, and also to identify the interpersonal conditions involved at the time of the acute episode’s emergence. Thus, the spatiotemporal dimension of the personal history allows the understandability of the interpersonal processes involved in schizophrenia from a broader perspective.

From the individual analysis of the life stories, it is possible to identify the patients’ biographical milestones, the personal meanings involved in their significant social interactions, and also continuity in their experience of vulnerability of being in the world, which are useful elements to consider for psychotherapeutic treatment.

The cross-sectional analysis of the life stories shows that a severe disorder of intersubjectivity starts in early adolescence, which should be a useful element to consider for the early detection and on the prevention. Beginning at an early stage, the patients progressively distance themselves from the social world, ending in a radical withdrawal. This distancing becomes a structural element, a key part of the prodromal stage of schizophrenia, as it was found in every case of the broader sample covering 15 patients with schizophrenia.

Social interactions are interrupted prior to the emergence of acute symptoms, possibly due to the threatening or anxiety provoking encounters with others. Nevertheless, the underlying anguish was not measured in this study. Instead, the study shows the personal vulnerability that leads to a psychotic break (or to the culmination of the intersubjective interruption).

### CLINICAL IMPLICATIONS

Psychotherapeutic interventions for patients with schizophrenia have been widely neglected in general. Current treatments are primarily with medication, including elctroconvulsive treatments in acute phases, thus following a medical-biological model that has not been questioned sufficiently. In this context, the intersubjective dimension seems extremely relevant for both the development of psychological treatments and the understanding of the interpersonal processes involved in schizophrenia (as an interruption in intersubjectivity).

From the very start of hospitalization, psychotherapeutic support would appear of fundamental importance. The patients should be accompanied on their return to intersubjectivity, whereas efforts should be made to provide proper emotional support for the realization of the overall problem affecting them. Prior to interventions focused on tasks (for example, successfully performing a social role, such as studying or working), the patients need to experience being in the world with another person, in a synchronous accompaniment of affective reciprocity.

In other words, the intersubjective dimension should be integrated in psychotherapeutic models focusing on the patients’ social interactions. These models should be oriented to developing a collaborative encounter between the patient and the therapist, as well as enhancing metacognitive capacities, as it has been shown to be helpful especially for the recovery of patients with schizophrenia in several case studies (Dimaggio et al., 2008; Harder and Folke, 2012; Lysaker et al., 2013).

The process of recovering understandability would be a key aspect in overcoming the patients’ alienation. Therefore, special consideration should be given to psychotherapeutic approaches that focus upon encouraging patients’ self-understanding and the establishment of a common communicative base between patient and psychotherapist (Holma and Aaltonen, 1997, 2004a,b; Seikkula and Olson, 2003; Seikkula et al., 2006). The idea is that the patient’s experience can be explicitly shared on the basis of a common meaning by a dialog process that takes into account the other’s point of view (or second person-perspective; Stanghellini and Lysaker, 2007).

Patients’ narrativity should improve along different levels of articulation, by the recognition of beliefs, the incorporation of emotions and the reconstruction of different meaningful life events. However, during acute phases delusional beliefs constitute the patients’ only available form of cognitive and interpersonal organization, so instead of confronting them, the focus should be placed on the difficulty in pragmatically comprehending others and on the experience of vulnerability (Lysaker et al., 2011a,b,c; Salvatore et al., 2012a,b; Henriksen and Parnas, 2013; Škodlar et al., 2013).

Besides, acute psychosis in schizophrenia manifests itself with a collapse of the temporal dimension of the narrative plot, which leads to a de-contextualization of self-experience (Holma and Aaltonen, 1997, 2004a; France and Uhlin, 2006). From the so called “literacy hypothesis” (Havelock, 1980, 1991), which belongs to studies that follow the transition from orality to literacy in the development of the thematic consciousness, it could be noted that in the acute phase the patients lose the modality of ordering their experience in consensual logical sequences, displaying a narrativity with epic or poetic characteristics (Guidano, 1999).

The re-establishment of the consensual ordering given by the locational/situational aspects of the life story (by articulating the self-experience in thematic/chronological sequences; Havelock, 1980, 1991; Bruner and Weisser, 1991; Narasimhan, 1991; Guidano, 1999; Irarrázaval, 2003; Bruner, 2004; Holma and Aaltonen, 2004a) allows to follow the patients’ progressive withdrawals from the social world, and also to identify the interpersonal conditions involved at the time of the acute episode’s emergence.

In this sense, the articulation of the patients’ life stories, expressed as narrative creations of their own subjectivity (and meanings), allows for the spatiotemporal dimension “re-ordering,” as well as for the understanding of the interpersonal processes involved in schizophrenia from a broader perspective. This psychological understanding reveals the intersubjective dimension that connects the emergence of the acute episode with the patients’ biographies, taking into account the personal meaning at play in each case.

In the case of Santi, there appears to be a need for emotional support aimed at accompanying him in becoming aware of the magnitude of the loss caused by the recent death of his father and, subsequently, to help him to develop strategies to deal with his feeling of abandonment in the world.

With Angel, his fear of ridicule is a structural emotional trait that dominates his life and is becoming a fundamental part of his worldview. Here, it is most important to deal with his sense of embarrassment and help him to accept himself. The aim is to provide a new, positive meaning to the sense of himself, overcoming his fear of ridicule in his encounters with others, or in other words, recovering the legitimacy of the sense of himself.

Salva requires an intervention in terms of developing a more basic sense of self-embodiment, which would be aimed at reflecting the feelings of “the other,” to re-establish primordial reciprocity. Additionally, space needs to be created in which the patient can recover a feeling of protection in the world, overcoming the feeling of powerlessness.

From this viewpoint, taking into consideration the story the patient tells of himself improves the articulation of self-narrative, which should gradually be extended toward diverse areas of his life whose elaboration appears important for him to make his way back to daily life. It would be important to articulate the present considering the experience that takes place in the actual interpersonal context, and from here to articulate the future as a horizon of possibilities.

Therefore, reconstructing the intersubjective dimension of the patients’ life stories shed light not only on the interpersonal processes involved in schizophrenia, but also on the psychotherapeutic intervention best suited to each individual case. Moreover, when intervention in acute phases of schizophrenia focuses mainly on reducing “positive” symptomatology, without assessing the psychological and social elements that are part of the overall situation affecting the patient, relapse seems highly likely.

### LIMITATIONS OF THE STUDY

Regarding the limitations of the study, mainstream scientific research in mental health has been dominated by quantitative methodologies and statistical analyses of big samples (representativeness), while the value of in-depth psychological analyses has been underestimated.

There is a predominant excessive confidence in the accuracy of numbers, as if they could not be easily manipulated in data analyses. This tendency has been supported by the illusion that numbers represent exactly (as a mathematical formula) the experience of the subject, rather than the patients’ own stories.

While qualitative methodology has been the tradition for research in humanities and social sciences, psychotherapy research has been developed using the methodologies of the medical sciences, which are mostly quantitative, being the randomized controlled trials being the favored design.

Nevertheless, research in psychotherapy should be guided by questions that are relevant to clinical practice. It should not be forgotten that methodologies are only means to carry out scientific research, but should not be the ultimate aim in themselves. Thus in this field of research it seems necessary to incorporate the questions psychotherapists need to answer to improve the practice of psychotherapy (to help patients), and then to choose the most appropriate methodologies.

However, one of the main advantages of qualitative studies is the open, mindful and detailed assessment of the subjective experience, enabling the emergence of the patients’ worldview and their personal meanings, which cannot be obtained by means of superficial assessments. Therefore, psychotherapists should also have a voice on the debate of which methodology is best suited to improving the practice of psychotherapy.

### FUTURE DIRECTIONS

Certainly, it would be important to systematize the results of this study in a model of psychotherapeutic treatment for persons with schizophrenia, which should include the intersubjective dimension, starting from the hermeneutic analysis of the patients’ life-worlds toward a meaning-based psychotherapeutic practice. This model would eventually require evidence of effectiveness.

Moreover, it would be interesting to explore gender differences in the processes involved in schizophrenia, investigating prodromal and acute stages, as well as life stories of women with schizophrenia. In addition, improvement is needed regarding the differential diagnosis between acute phases of schizophrenia and acute phases of other severe mental disorders, such as major depression and bipolar disorder.

Finally, the future challenge in the field of phenomenological psychopathology would be to develop a comprehensive/unified philosophical framework for an embodied science of intersubjectivity. And, consistently, to continue developing coherent methodologies for empirical research, since this is the closest we can get to the patients’ life-worlds.

## AUTHOR CONTRIBUTIONS

Co-author Dariela Sharim made substantial contributions to the analysis and interpretation of data to include in the paper; she revised the paper critically for important intellectual content; she made a final approval of the actual version of the paper to be published; she agreed upon the accuracy and coherence of the development of the sections for the paper.

## Conflict of Interest Statement

The authors declare that the research was conducted in the absence of any commercial or financial relationships that could be construed as a potential conflict of interest.
